# Clinical and molecular characterization of craniofrontonasal syndrome: new symptoms and novel pathogenic variants in the *EFNB1* gene

**DOI:** 10.1186/s13023-021-01914-1

**Published:** 2021-06-26

**Authors:** Ewelina Bukowska-Olech, Paweł Gawliński, Anna Jakubiuk-Tomaszuk, Maria Jędrzejowska, Ewa Obersztyn, Michał Piechota, Marta Bielska, Aleksander Jamsheer

**Affiliations:** 1 Department of Medical Genetics, Poznan University of Medical Sciences, Rokietnicka 8 Street, 60-806 Poznan, Poland; 2 Department of Medical Genetics, Institute of Mother and Child, Warsaw, Poland; 3 Department of Pediatric Neurology and Rehabilitation, Medical University of Bialystok, Bialystok, Poland; 4 Medical Genetics Unit, Mastermed Medical Center, Bialystok, Poland; 5 Department of Medical Genetics, The Children’s Memorial Health Institute, Warsaw, Poland; 6 Centers for Medical Genetics GENESIS, Poznan, Poland; 7 Department of Pediatrics, Hematology, Oncology and Diabetology, Medical University of Lodz, Lodz, Poland

**Keywords:** Monozygosity, Discordant phenotype, *EFNB1*, Ephrin B1, Coronal craniosynostosis, Custom targeted next-generation sequencing

## Abstract

**Background:**

Craniofrontonasal syndrome (CFNS) is a rare X-linked disorder that results from pathogenic variants in the *EFNB1* gene. The syndrome paradoxically presents with greater severity of the symptoms in heterozygous females than hemizygous males.

**Results:**

We have recruited and screened a female cohort affected with CFNS. Our primary finding was the description of monozygotic twins, i.e., patients 5 and 6, discordant for the CFNS phenotype. Intriguingly, patient 5 presented classical CFNS gestalt, whereas patient 6 manifested only very subtle craniofacial features, not resembling CFNS. Besides, we have expanded the mutational spectrum of the *EFNB1* gene through reporting four novel pathogenic variants—p.(Trp12*), p.(Cys64Phe), p.(Tyr73Metfs*86), p.(Glu210*). All those alterations were found applying either targeted NGS of a custom gene panel or PCR followed by Sanger sequencing and evaluated using in silico predictors. Lastly, we have also expanded the CFNS phenotypic spectrum by describing in patient 3 several novel features of the syndrome, such as bifid hallux, bicornuate uterus, and abnormal right ovary segmented into six parts.

**Conclusions:**

We have described the unreported so far differences of the clinical phenotype in the monozygotic twin patients 5 and 6 harboring an identical p.(Glu210*) variant located in the *EFNB1* gene. With our finding, we have pointed to an unusual phenomenon of mildly affected females with CFNS, who may not manifest features suggestive of the syndrome. Consequently, this study may be valuable for geneticists consulting patients with craniofacial disorders.

**Supplementary Information:**

The online version contains supplementary material available at 10.1186/s13023-021-01914-1.

## Introduction

Craniofrontonasal syndrome (CFNS; MIM: 304110) is a rare X-linked disorder that inherits in a paradoxical manner, exceptionally presenting greater severity of symptoms in heterozygous females than hemizygous males [1, 2]. The clinical picture in the affected females encompasses coronal craniosynostosis (CS), frontal bossing, hypertelorism, depressed nasal bridge, bifid nose, craniofacial asymmetry, downslanting palpebral fissures, frizzy and curly hair, syndactyly and longitudinally ridged fingernails. Intriguingly, many symptomatic hemizygous men show merely hypertelorism with no other congenital anomalies or major facial dysmorphism [3, 4].

Wieacker and Wieland in 2005 explained the above paradox as a cellular interference, which assumes that due to a random X-inactivation, heterozygous females are uniquely mosaic and therefore have both functional and nonfunctional ephrin-B1, a protein which is encoded by the *EFNB1* gene [5]. These two ephrin-B1 forms’ coexistence affects the adhesion and sorting of cells, disrupting normal embryological development [6, 7]. Further reports describing more severely affected males, who all were mosaic for deleterious variants in the *EFNB1*, strengthen the hypothesis about the described pathomechanism’s biological relevance [8]. However, the precise molecular explanation for this phenomenon remains not yet fully understood [7].

## Cohort description

We recruited four sporadic female individuals (patients 1–4) and one familial case consisting of two female individuals (patient 5 and 6), out of whom all but one, i.e., patient 6, presented phenotypic features suggestive of CFNS.

## Methods

### Targeted next-generation sequencing NGS

We designed and applied the custom On-Demand AmpliSeq (ThermoFisher Scientific) panel targeting 37 genes related to craniofacial disorders [9, 10]. We constructed the barcoded gDNA libraries according to the manufacturer’s sample preparation protocol (Ion AmpliSeq Library Kit 2.0; On-Demand Panels) and subsequently sequenced them on the Ion Torrent S5 platform using the Ion 530™ or 540™ Chip.

### PCR and Sanger sequencing

PCR followed by Sanger sequencing was used to validate variants detected through targeted NGS (patient 1 and 5) and screen the coding sequence of the *EFNB1* gene (patients 2–4). Besides, we performed targeted Sanger sequencing in the twin sister of patient 6 (targeted analysis of exon 4). We designed specific primers (Additional file 1: Table S1) using Primer3 tool v. 0.4.0. The PCR reactions and PCR product purifications were carried out following standard protocols. Next, Sanger sequencing was performed on an automated sequencer Applied Biosystems Prism 3700 DNA Analyzer using dye-terminator chemistry kit v.3, ABI 3130XL. Finally, the variant was visualized by applying the BioEdit tool and annotated against the reference *EFNB1* sequence NM_004429.4 following the Human Genome Variation Society (HGVS) nomenclature guidelines.

### Zygosity test

We used Devyser Complete v2 kit (Devyser, Sweden) following the manufacturer’s protocol to analyze the twin sisters zygosity status (patient 5 and 6). The kit contains 33 short tandem repeats (STRs) markers localized on 13, 18, 21, X and Y chromosomes.

### X chromosome inactivation (XCI) assay

We performed an XCI assay based on the methylation specificity of restriction enzymes at STRs located within the *AR* gene (patient 5 and 6). We used *Hpa*II restriction endonuclease that presents a particular activity only on unmethylated gDNA. 100 ng of gDNA was digested with either 20 U *Rsa*I (reference sample) or a mixture of enzymes, i.e. 20 U *Rsa*I and 20 U *Hpa*II (Thermo Fisher Scientific). After incubation and inactivation of enzymes, we performed PCR amplification. The reaction was set up using FAM-labeled primers 5′-TCCAGAATCTGTTCCAGAGCGTGC-3 (forward), 5′-GCTGTGAAGGTTGCTGTTCCTCAT-3 (reverse) as described by Janczar et al. [11, 12]. We separated the PCR products on an ABI 3130 DNA sequencing analyzer (Applied Biosystems) and analyzed in GeneMarker software v2.7.0 (SoftGenetics). The area under the peak was calculated and normalized [11].

### Face2Gene analysis

We used the Face2Gene tool to test whether the craniofacial symptoms present in twin patients 5 and 6 were characteristic of CFNS. Face2Gene’s inbuilt algorithm quantifies facial gestalt based on hundreds of photographs of specific and confirmed syndrome patients. As a result, a list of possibly matching syndromes is created and ranked with a score called Gestalt Score.

## Results

### Clinical report

We recruited six female cases, out of whom all but one, i.e., patient 6, presented with phenotypic characteristics suggestive for CFNS. The comparison of all clinical features noted in our cohort was outlined in Table 1. The extended clinical description was presented in what follows only for those cases with either additional or unusual CFNS manifestation (patients 3, 5 and 6).

**Table 1:** Clinical manifestations of seven patients with craniofrontonasal syndrome

#	Features	HPO no.	Patient 1	Patient 2	Patient 3	Patient 4	Patient 5	Patient 6
1	Variant: NM_004429.4		c.35G>A	c.191G>T	c.216del	c.451G>A	c.628G>T	c.628G>T
2	Sex		F	F	F	F	F	F
3	Relationship		na	na	na	na	Twin 1	Twin 2
4	Hypertelorism	HP:0000316	+	+	+	+	+	−
5	Epicanthus	HP:0000286	−	+	+	−	+	−
6	Down-slanting palpebral fissures	HP:0000494	Up-slanting palpebral fissures	−	Up-slanting palpebral fissures	+	−	−
7	Anteverted nares	HP:0000463	+	+	+	+	+	+
8	Depressed nasal bridge	HP:0005280	+	+	Prominent nasal bridge	+	+	+
9	Midline nasal groove	HP:0004112	+		+	+	+	−
10	Abnormality of the pinna	HP:0000377	+ thick helix	−	Prominent antihelix	+	+	−
11	Low-set ears	HP:0000369	−	+	+	+	+	+
12	Coarse facial feature	HP:0000280	+	+	+	+	+	+
13	Midface retrusion	HP:0011800	+	+	+	+	−	−
14	Micrognathia	HP:0000347	+	−	−	+	+	+
15	High palate	HP:0000218	+	+	+	+	−	−
16	Anterior open bite	HP:0200095	+	+	+	+	−	−
17	Cleft upper lip	HP:0000204	−	−	−	−	−	−
18	Bilateral cleft lip and palate	HP:0002744	−	−	−	−	−	−
19	Ankyloglossia	HP:0010296	−	?	+	−	−	−
20	Hoarse voice	HP:0001609	−	?	+	−	*	*
21	Short neck	HP:0000470	+	+	+	+	−	+
22	Small anterior fontanelle	HP:0000237	−	?	?	?	−	−
23	Dysgenesis of the corpus callosum	HP:0006989	?		+ (posterior part)	−	?	?
24	Agenesis of the corpus callosum	HP:0001274	?	+	−	+	?	?
25	Plagiocephaly	HP:0001357	−	+	−	+	+	
26	Craniosynostosis	HP:0001363	−	+	+	+	+	−
27	Global developmental delay	HP:0001263	+ Mild ID	−	+ Mild ID	+	−	−
28	Brachydactyly	HP:0001156	+	−	+	−	+	+
29	Broad thumb	HP:0011304	+	−	Duplicated thumb	−	+	−
30	Toe syndactyly Finger syndactyly	HP:0001770 HP:0006101	−	+ (feet)	−	−	+ (feet)	−
31	Longitudinal ridging of toenails	HP:0001807	+	+	+	?	+	+
32	Longitudinal ridging of fingernails	HP:0001807	+	+	+	+	+	+
33	Shoulder girdle muscle atrophy	HP:0003724	−	+	+	?	*	*
34	Limited shoulder movement	HP:0006467	−	?	+	−	*	*
35	Low-set nipples	HP:0002562	+	+	+	−	−	−

ID, intellectual disability; HPO no., Human Phenotype Ontology database number identification for phenotypic abnormality [22]; Symbols: +, feature present; (+); −, feature absent; nd, no data; na, not applicable

* – the symptom cannot be assessed (the patient too young)

## Patient 3

**Fig. 1:**
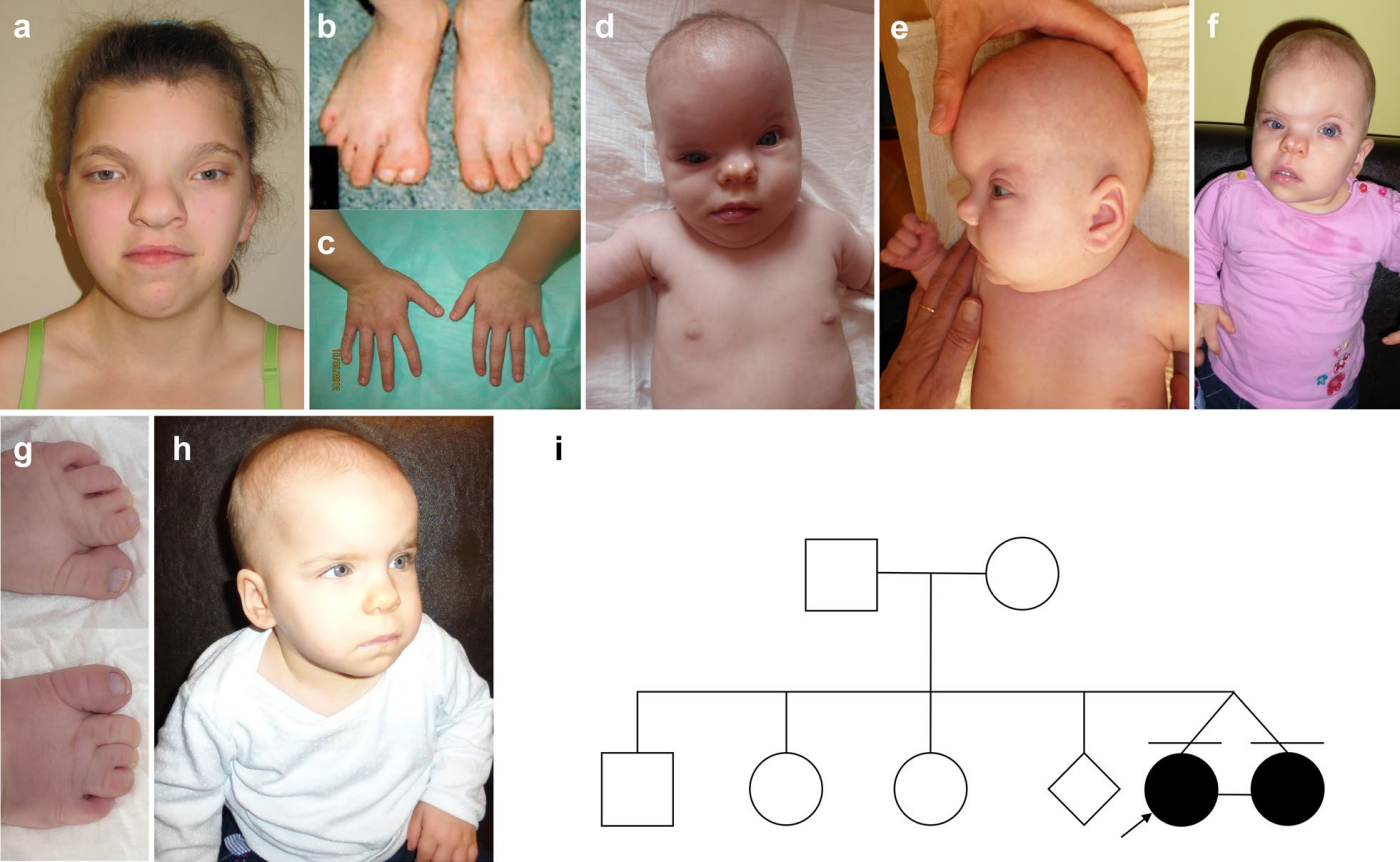
Patient 3 **a** manifested hypertelorism, up-slanted palpebral fissures, anteverted nares, prominent nasal bridge and overall impression of coarse facial features. She presented also partial skin syndactyly of toes 2–3, bifid hallux and clinodactyly of the 5th fingers (**b**, **c**). Patient 5 and patient 6 are monozygotic twin sisters with a highly variable clinical presentation of CFNS. While patient 5 presents with typical facial features of CFNS (**d**–**g**), patient 6 (**h**) shows a relatively mild phenotype (see Table 1 for details) that is not suggestive of CFNS. The family pedigree of monozygotic twins (**f**). The analysis of relatives was not possible because twins were adopted

Patient 3 was a girl born in the 40th week of gestation from 8th pregnancy to unrelated healthy parents (Fig. 1a–c). The pregnancy history was unremarkable. Her body mass was 5100 g (> 97th percentile), length 59 cm (> 97th percentile), Apgar score was 8–10–10 at 1′, 3′ and 5′. In addition to classical CFNS symptoms, she presented also a bifid hallux (Fig. 1a–c) bicornuate uterus, abnormal right ovary segmented into six parts by five serpentine-like constrictions, with the largest ovary part of 1.5 cm, while the smallest one of 0.5 cm in diameter. She also showed congenital horizontal nystagmus, alternating divergent strabismus, defects of the genitourinary system, including horseshoe kidney. MRI revealed hypoplastic corpus callosum, whereas cerebellum and brainstem were unchanged. All this features have not been noted among patients affected with CFNS thus far.

## Patient 5

Patient 5 was a girl born in the 38th week of gestation from 4th pregnancy twined to unrelated healthy parents (Fig. 1i). The pregnancy history was unknown because patient 5 was adopted. Her body mass was 2370 g (< 3rd percentile), length 49 cm (< 3rd percentile), Apgar score was 8–10–10 at 1’, 3’ and 5’. She was referred for dysmorphic evaluation at 1st month of age. She had a coarse face, plagiocephaly, CS, micrognathia, a small anterior fontanel, significant hypertelorism, bilateral epicanthal folds, bilateral low-set ears, flat nasal bridge, anteverted nares, and a midline crease of the nasal tip. Brachydactyly, syndactyly of toes and longitudinal ridging of a finger- and toenails were also observed (Fig. 1d–g). On examination at the age of 5.5 months, she presented with a weight of 6110 g (< 3rd centile) and head circumference of 37.8 cm (< 3rd centile).

## Patient 6

Patient 6 was a girl born in the 38th week of gestation from 4th pregnancy twined to unrelated healthy parents (Fig. 1i). The pregnancy history was unknown because patient 6 was adopted. Her body mass was 2330 g (< 3rd percentile), length 50 cm (< 3rd percentile), Apgar score was 8–8–9 at 1’, 3’, 5’ and 10’. She was referred for dysmorphic evaluation at 4th month of age since her twin sister obtained a diagnosis of CFNS. She had mild coarse facial features, anteverted nares, depressed nasal bridge, short neck and longitudinal ridging of fingernails and toenails (Fig. 1h).

## Targeted NGS and Sanger sequencing

**Fig. 2:**
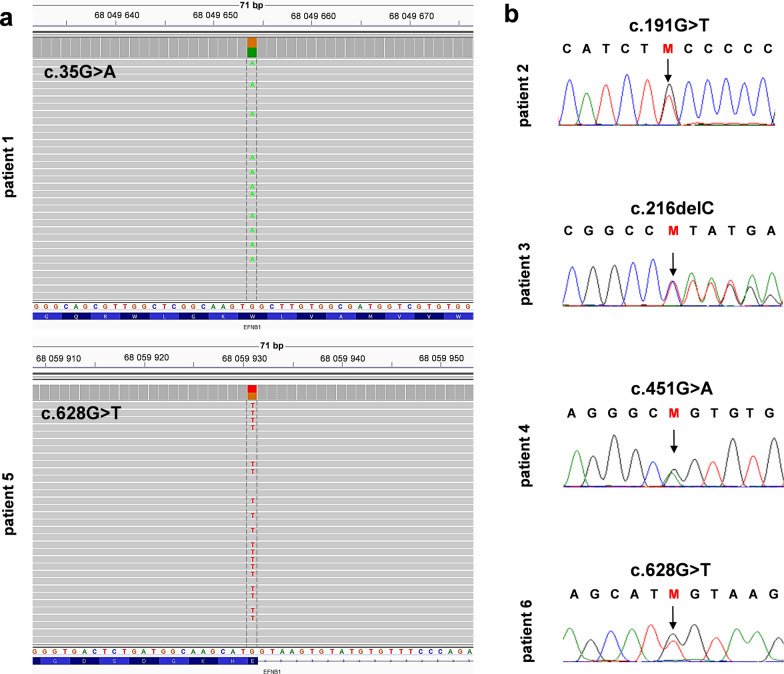
Targeted next-generation sequencing results (**a**). Pathogenic single-nucleotide variants in the *EFNB1* gene were visualized using Integrative Genomics Viewer (IGV)—c.35G>A p.(Trp12*) in patient 1 and c.628G>T p.(Glu210*) in patient 5. Targeted Sanger sequencing of the *EFNB1* gene results (**b**). Pathogenic single-nucleotide variants in the *EFNB1* gene were visualized using BioEdit tool—c.191G>T p.(Cys64Phe) in patient 2, c.216del p.(Tyr73Metfs*86) in patient 3, c.451G>A p.(Gly151Ser) in patient 4 and c.628G>T p.(Glu210*) in patient 6

**Fig. 3:**
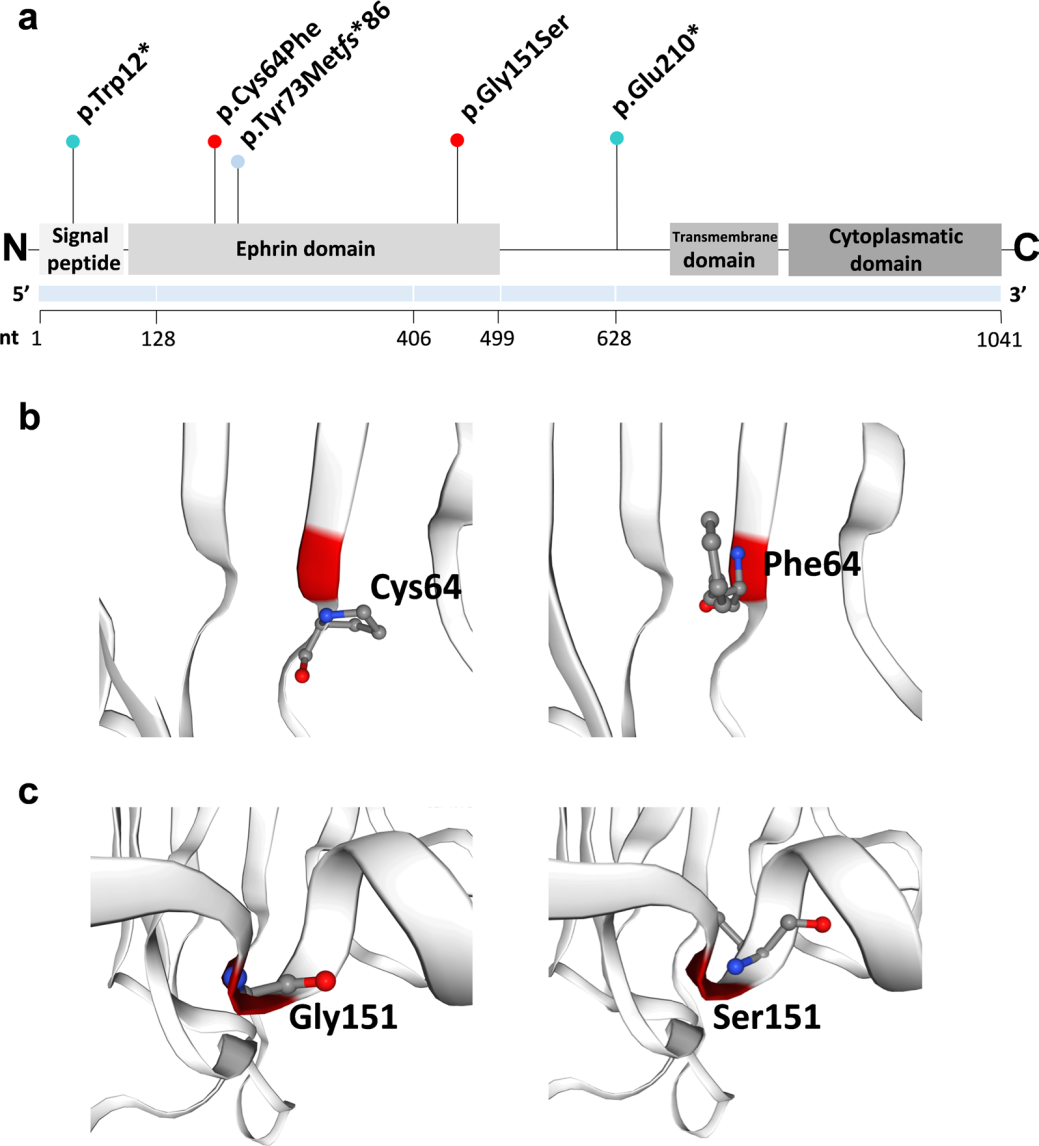
Schematic view of the *EFNB1* gene and ephrin-B1 structure with an overview of all single nucleotide variants identified in this study (**a**). Ephrin-B1 is encoded by the *EFNB1* gene and consists of four structural units, such as a signal peptide, ephrin, transmembrane and cytoplasmatic domains. Similarly to our results, the great majority of all pathogenic variants occurs within the first three exons and are expected to disrupt the signal peptide and the ephrin domain of ephrin-B1. The 3D visualization of both wild type and mutated missense alterations in the ephrin-B1 made applying SWISS-MODEL, i.e., p.(Cys64Phe) (**b**) and p.(Gly151Ser) (**c**)

gDNA (isolated from peripheral blood lymphocytes) of Patients 1 and 5 was subject to targeted NGS of a custom gene panel that revealed two novel heterozygous variants in the *EFNB1* gene—c.35G>A p.(Trp12*) and c.628G>T p.(Glu210*), respectively (Fig. 2a). The presence of both alterations was confirmed by Sanger sequencing. Patients 2–4 were screened before the advent of the NGS method. Thus, the molecular diagnosis was achieved by Sanger sequencing on gDNA isolated from peripheral blood lymphocytes, which revealed the presence of the following three heterozygous alterations out of which two were novel—c.191G>T p.(Cys64Phe), c.216del p.(Tyr73Metfs*86). In contrast, one variant has been previously reported c.451G>A p.(Gly151Ser) (HGMD no: CM041297) (Fig. 2b). The family history of patient 5 showed that she has a twin sister who, despite the lack of typical CFNS symptoms, underwent targeted PCR and Sanger sequencing. We evaluated the pathogenicity of missense variants in silico applying multiple online prediction tools including Polyphen-2, SIFT, CADD, MutationTaster and other resources such as DANN, FATHMM-MKL, LRT, BayesDel addAF, BayesDel noAF, GERP, PhyloP100, PhastCons integrated into either VarSome online tool or Alamut® Visual software product. The classification of all variants was performed following the American College of Medical Genetics and Genomics (ACMG) guidelines (Table 2). Applying SWISS-MODEL, we have visualized in 3D both wild type and mutated missense alterations in the ephrin-B1, i.e., p.(Cys64Phe) and p.(Gly151Ser) [13] (Fig. 3).

**Table 2:** The overview of missense and nonsense variants found in the *EFNB1* gene analyzed through MutationTaster, Varsome online tools (obtained on 2th November 2020) and Alamut® Visual software (obtained on 10th November 2020)

	Patient 1	Patient 2	Patient 4	Patient 5 and 6
coding DNA level (NM_004429.4)	c.35G>A	c.191G>T	c.451G>A	c.628G>T
gDNA level	g.815G>A	g.9683	g.10712	g.11092
chromosomal level (GRCh38)	chrX:68829811G>A	chrX:68838679G>T	chrX:68839708G>A	chrX:68840088G>T
Protein level (NP_004420.1)	p.Trp12*	p.Cys64Phe	p.Gly151Ser	p.Glu210*
Exon	1	2	3	4
HGMD (v15.11) no.	Not reported	Not reported	CM041297	Not reported
dbSNP rs no.	rs1482772814	Not reported	rs28936069	Not reported
gnomAD (v2.1.1)	Not reported	Not reported	Not reported	Not reported
1000 Genomes	Not reported	Not reported	Not reported	Not reported
ACMG classification	Pathogenic	Likely pathogenic	Likely pathogenic	Pathogenic
SIFT (v6.2.0)	n.d.	Deleterious	Deleterious	n.d.
PolyPhen-2 (v2)	n.d.	Probably damaging	Probably damaging	n.d.
DANN (v2014)	0.9954	0.9935	0.9989	0.9969
FATHMM-MKL (dbNSFP v4.1)	Damaging	Damaging	Damaging	Damaging
LRT (dbNSFP v4.1)	Neutral	Deleterious	Deleterious	Neutral
BayesDel addAF (v4.1)	Damaging	Damaging	Damaging	Damaging
BayesDel noAF (v4.1)	Damaging	Damaging	Damaging	Damaging
MutationTaster (v2013)	Disease causing	Disease causing	Disease causing	Disease causing

### Zygosity analysis

The monozigosity of twin patient 5 and 6 was confirmed based on an analysis of 33 STR markers localized on 13, 18, 21, X and Y chromosomes.

### XCI

We detected random XCI in twin patient 6 (46% vs. 54%), who manifested facial features unsuggestive for CFNS, whereas non-random XCI (84% vs. 16%) in twin patient 5, who showed a classical CFNS facial phenotype.

### Face2Gene analysis

The craniofacial phenotype of patient 6 was assessed using Face2Gene online available tool. Among the suggested 30 different syndromes, CFNS was not listed by the algorithm. However, the first five proposed diagnoses were as follows—Cornelia de Lange syndrome, Costello syndrome, Megalencephaly-Capillary Malformation-Polymicrogyria Syndrome, Alpha-Thalassemia/mental Retardation Syndrome and CHARGE syndrome. On the contrary, the phenotype of patient 5 was correctly identified as CFNS (listed as second).

## Discussion

Although monozygotic twins originate from a single zygote and share the same genetic material and similar intrauterine environment, they occasionally may show discordant phenotypes of monozygotic disorder. The differences in clinical phenotype can be explained through at least several mechanisms such as epigenetic factors, an asymmetric split of the embryo, discordant cell differentiation or abnormalities in placental blood flow [14–16]. Intriguingly, our primary finding was the evaluation of monozygotic twin patients, i.e. patient 5 and 6, who presented with highly variable severity of the CFNS symptoms. Both individuals carried the same p.(Glu210*) pathogenic *EFNB1* variant and identical germline genetic information. In patient 5, we noted a typical female presentation of CFNS (Table 1; Fig. 1a–d). In contrast, in patient 6, we only detected mild facial anomalies unsuggestive for CFNS, including anteverted nares, depressed nasal bridge, low-set ears, coarse facial features, micrognathia and short neck (Table 1; Fig. 1e). Besides, the craniofacial phenotype of patient 6 was analyzed using Face2Gene, which did not match CFNS among the possible dysmorphological diagnoses.

Mild clinical features in female individuals with CFNS are rather unusual. As mentioned before, CFNS inherits paradoxically and presents more severe clinical symptoms in females, who harbour the heterozygous *EFNB1* variants in comparison to hemizygous males. Furthermore, rarely reported mosaic male individuals are more severely affected than their hemizygous counterparts. This is because other ephrin family members can presumably substitute the complete lack of ephrin-B1 in purely hemizygous males [3, 7, 17]. In the medical literature, we have found merely one description of mildly affected CFNS female patient. Twigg et al. reported a familial case (family no. 3217) heterozygous for a missense pathogenic variant p.(Pro54Leu), in which one of the affected females had minimal clinical manifestations of CFNS. However, this patient was shown to have a lower mutation level in the hair roots and buccal swab [2]. In our case, we were unable to check for the mosaicism in mesoderm or ectoderm-derived cell lines, although the level for the causative variant in blood cells reached 50% of reads, being unsuggestive of somatic mosaicism.

Except for mosaicism in other than blood cells, one may suspect the presence of additional modifiers of the phenotype, including epigenetic factors [18–20]. To check whether the variable severity of CFNS in both twin females resulted from skewed X chromosome inactivation, we performed XCI testing. We hypothesized that similar to male patients who show minimal CFNS symptoms, our mildly affected twin sister may have a highly preferential expression of the *EFNB1* from a single gene copy, resembling its status in hemizygosity. To our surprise, we demonstrated unequal XCI in the severely affected twin patient 5 (84% vs. 16%) and almost random X inactivation in the mildly affected twin patient 6 (46% vs. 54%). Our finding, therefore, suggests that skewed X inactivation cannot account for the mild presentation of CFNS in one of our twin sisters and probably other mildly affected female individuals. Recently, another research group did not find evidence for preferential XCI or a distinct correlation between XCI ratios in a group of familial X-linked hypohidrotic ectodermal dysplasia patients showing variable disease manifestation [21]. Hence our result strengthens the above conclusion regarding the presence of additional yet undetected modifying factors resulting in discordant phenotype in X-linked disorders.

Second, we have also compared the phenotypic presentation of the six female CFNS individuals (Table 1). We have noticed that all patients, except for patient 6, manifested the following clinical features—hypertelorism, CS (also except for patient 1), low set ears, coarse facial features, high palate, anterior open bite and longitudinal riding of the fingernails. The rarest clinical feature was brachydactyly (patient 3; Fig. 1). Our observation may suggest that CNFS has a relatively constant set of features. However, we have also broadened the phenotypic spectrum of CFNS syndrome, as we reported new features present in patient 3, such as a bifid hallux, bicornuate uterus and abnormal right ovary segmented into six parts.

Lastly, we have expanded the *EFNB1* gene mutational spectrum as we described three additional novel variants located in the *EFNB1* gene—p.(Trp12*), p.(Tyr73Metfs*86), p.(Glu210*) and consequently increased the total number of CFNS-associated pathogenic variants to 123. All newly identified alterations were found applying either targeted NGS of a custom gene panel or PCR followed by Sanger sequencing. Subsequently, we evaluated the pathogenicity of the detected variants using in silico predictors (Table 2).

## Conclusions

First of all, we have pointed to an unusual phenomenon of mildly affected females with CFNS, who may not manifest features suggestive of the syndrome. As a consequence, this study may be valuable for clinical geneticists consulting patients with craniofacial disorders and who potentially may overlook such individuals. Second, we excluded skewed XCI pattern as a cause of discordant phenotype in monozygotic twins described here. Our study strengthens the recent conclusion regarding the presence of additional yet undetected modifying factors resulting in X-linked disorders’ discordant phenotype. Third, we have also expanded the CFNS phenotypic spectrum by describing in patient 3 novel features of the syndrome, such as bifid hallux, bicornuate uterus, and abnormal right ovary segmented into six parts. Finally, we have expanded the mutational spectrum of the *EFNB1* gene by reporting three other novel pathogenic variants causing CFNS.

## Supplementary Information

**Additional file 1**: Table 1: List of primers used for PCR and Sanger sequencing.

## Data Availability

The datasets for this article are not publicly available due to concerns regarding participants/ patients anonymity. Requests to access the datasets should be directed to the corresponding author.

## Abbreviations

ACMG: American College of Medical Genetics and Genomics
CFNS: Craniofrontonasal syndrome
CS: Craniosynostosis
NGS: Next-generation sequencing
XCI: X chromosome inactivation

## Declarations

### Web resources

ClinVar: https://www.ncbi.nlm.nih.gov/clinvar/. Face2Gene: https://www.face2gene.com/. gnomAD: https://gnomad.broadinstitute.org/. HGMD: http://www.hgmd.cf.ac.uk/ac/index.php. OMIM: https://www.omim.org/. Primer3: http://bioinfo.ut.ee/primer3-0.4.0/. Varsome: https://varsome.com/.
