# Supplementary material for: Clinical and molecular characterization of craniofrontonasal syndrome: new symptoms and novel pathogenic variants in the EFNB1 gene
Source: Orphanet J Rare Dis. 2021 Jun 26;16:286. doi: 10.1186/s13023-021-01914-1 (PMC8236199; doi:10.1186/s13023-021-01914-1)
Supplement: Supplementary file 1 — Additional file 1: Table 1: List of primers used for PCR and Sanger sequencing. [file 13023_2021_1914_MOESM1_ESM.docx]

**Table 1** List of primers used for PCR and Sanger sequencing

| **Exon** | **Forward primer sequence 5’-3’** | **Reverse primer sequence 5’-3’** |
| --- | --- | --- |
| 1 | ggcagaggaaggcgagg | acctccccacatgcactc |
| 2 | cttccctggttctggaatgg | tgcaccacttagaagctccc |
| 3 and 4 | tgggagtttctgggtaatgc | cagcttgcatttcttcaggg |
| 5a | gcgaaaggagaggtccg | ctgaagaaatgcaagctggg |
| 5b | ctcttttccagaccaaaccc | cacacactcagggattttgag |
